# Evaluation of Appropriate Reference Genes for Gene Expression Normalization during Watermelon Fruit Development

**DOI:** 10.1371/journal.pone.0130865

**Published:** 2015-06-25

**Authors:** Qiusheng Kong, Jingxian Yuan, Lingyun Gao, Liqiang Zhao, Fei Cheng, Yuan Huang, Zhilong Bie

**Affiliations:** Key Laboratory of Horticultural Plant Biology, Ministry of Education, College of Horticulture and Forestry Sciences, Huazhong Agricultural University, Wuhan, China; Northwestern University, UNITED STATES

## Abstract

Gene expression analysis in watermelon (*Citrullus lanatus*) fruit has drawn considerable attention with the availability of genome sequences to understand the regulatory mechanism of fruit development and to improve its quality. Real-time quantitative reverse-transcription PCR (qRT-PCR) is a routine technique for gene expression analysis. However, appropriate reference genes for transcript normalization in watermelon fruits have not been well characterized. The aim of this study was to evaluate the appropriateness of 12 genes for their potential use as reference genes in watermelon fruits. Expression variations of these genes were measured in 48 samples obtained from 12 successive developmental stages of parthenocarpic and fertilized fruits of two watermelon genotypes by using qRT-PCR analysis. Considering the effects of genotype, fruit setting method, and developmental stage, geNorm determined *clathrin adaptor complex subunit* (*ClCAC*), *β-actin* (*ClACT*), and *alpha tubulin 5* (*ClTUA5*) as the multiple reference genes in watermelon fruit. Furthermore, *ClCAC* alone or together with *SAND family protein* (*ClSAND*) was ranked as the single or two best reference genes by NormFinder. By using the top-ranked reference genes to normalize the transcript abundance of *phytoene synthase* (*ClPSY1*), a good correlation between lycopene accumulation and *ClPSY1* expression pattern was observed in ripening watermelon fruit. These validated reference genes will facilitate the accurate measurement of gene expression in the studies on watermelon fruit biology.

## Introduction

Watermelon (*Citrullus lanatus*) is a popular and economically important horticultural crop in terms of production and consumption. During development and ripening, watermelon fruits undergo various physiological and biochemical changes, resulting in diverse sizes, colors, shapes, sweetness, textures, and aromas [1]. Watermelon is gaining popularity as a model plant for studying non-climacteric fruits. Studies have focused on the unique metabolic and regulatory networks of watermelon fruit to improve the crucial nutritional attributes of this fruit [2]. Moreover, the vast amounts of genomic resources are beneficial for elucidating the biological processes involved in fruit development. Transcriptome sequencing has been conducted to study the transcriptional regulatory networks in watermelon fruit, revealing many key genes involved in sugar, citrulline, and carotenoid metabolisms [1, 3–5]. Target gene expression patterns during fruit development can provide clues to understand its biological functions. Real-time quantitative reverse-transcription PCR (qRT-PCR) is an efficient method to measure gene transcript abundance and to validate gene expression changes detected in RNA-Seq [1, 6] and microarray [2].

In qRT-PCR analysis, gene expression is quantified by normalizing to one or more internal reference genes presumed to be stable throughout a given experiment. Normalization removes considerable non-biological variations associated with the different steps of sample preparations and expression measurements [7]. Accordingly, qRT-PCR accuracy is strongly influenced by internal reference gene stability. The use of unstable reference genes in relative quantification of gene expression could lead to significant biases and misinterpretations of data [8–10]. Moreover, reference genes are not constantly expressed under a wide range of experimental conditions [11, 12]. Thus, systematic validation of reference genes must be conducted on each experiment prior to their use [13, 14]. Statistical algorithms such as geNorm [15] and NormFinder [16] have been developed to determine gene expression stability, which greatly simplified the choice of appropriate reference genes for normalization in qRT-PCR analysis.

Suitable reference genes have been identified in watermelon under normal growth conditions as well as biotic and abiotic stresses, providing a good starting point for reference gene selection in qRT-PCR analysis [17]. However, the reference genes in watermelon fruit have not been well characterized in this study. Considering all the organs and tissues under normal growth conditions, at least nine reference genes are required for accurate normalization in watermelon [17]. The use of numerous reference genes is not feasible in practice, particularly when the target genes are few. Meanwhile, an increasing number of evidence shows that reference genes are not universally applicable [12, 18]. Thus, the suitability of candidate reference genes must be carefully evaluated for each experimental system [19]. Stable reference genes have been specifically identified for the fruits of several crops, such as papaya [20] and blueberry [21]. To date, the single, non-validated *18SrRNA* has been used as reference gene in qRT-PCR analyses of watermelon fruit [1, 2, 22]. Considering that fruit development is a very complex process, the selection of appropriate reference genes that are not affected by developmental processes is required to study gene expression during fruit development. The identification of the fruit-specific reference genes for watermelon is critical because of its significance.

Fertilization and parthenocarpy are two different approaches for fruit setting that involves distinct gene expressions and regulatory systems during fruit development [23]; thus, they provide good opportunities for selecting reference genes with intrinsic expression stability during fruit development and ripening. In this study, the expression stability of 12 candidate reference genes was evaluated and compared across 48 cDNA samples obtained from 12 developmental stages of parthenocarpic and fertilized fruits of two watermelon genotypes. The appropriate reference genes, which were specific for normalizing the target genes in watermelon fruit by qRT-PCR analysis, were selected using geNorm and NormFinder algorithms.

The carotenoid content is one of the key characteristics to determine the fruit quality of watermelon. Lycopene is the dominant carotenoid in red-fleshed watermelon cultivars, contributing to the red color of watermelon flesh [1]. Phytoene synthase (PSY) catalyzes the first step in carotenoid biosynthetic pathway and is one of the key enzymes that regulate carotenoid contents in watermelon fruits. Two members of PSY gene family, namely, *ClPSY1* (Cla009122) and *ClPSY2* (Cla005425), were identified by transcriptome analysis; however, only *ClPSY1* was associated with the lycopene accumulation in watermelon fruits [1, 5]. Thus, the reliability of the identified reference genes was further validated by normalizing *ClPSY1* expression and testing its correlation with lycopene accumulation during fruit ripening. The validated reference genes will improve the accuracy of gene expression studies in watermelon fruits.

## Materials and Methods

### Plant materials and treatments

Watermelon (*C*. *lanatus*) inbred line 97103 and commercial F_1_ hybrid 8424 were used in the present study. Both were mature at 30 days after pollination and had red flesh. Seedlings at the third true-leaf stage were transplanted in a plastic greenhouse at Huazhong Agricultural University, Wuhan, China (east longitude 113°41′–115°05′, north latitude 29°58′–31°22). Fertilizer application and pest control were implemented following the standard commercial practices. Parthenocarpic fruits were induced by the application of *N*-(2-chloro-4-pyridyl)-*N*’-phenylurea (CPPU). One day before anthesis, the female flowers were isolated with paper bags to prevent natural pollination. On the day of anthesis, the paper bags were removed, and CPPU (Shiteyou, China) was applied by spraying unpollinated ovaries at a concentration of 10 μM in the morning following the manufacturer’s instruction. Afterward, the female flowers were covered again until the success of fruit set. Simultaneously, artificial pollination was performed on other female flowers by hand. The pollinated and CPPU-treated female flowers were tagged, and only one fruit per plant was allowed to develop. Fruits were harvested throughout the process of fruit development at 0, 1, 3, 5, 7, 9, 12, 18, 23, 27, 30, and 35 days after anthesis (DAA). Three fruits were randomly harvested at each sampling point, and each fruit represented a biological replication. The ovaries were sampled at 0, 1, and 3 DAA. Beyond 3 DAA, the harvested fruits were cut longitudinally, and flesh tissue was obtained from the center of the fruit. All the samples were immediately frozen in liquid nitrogen and then stored at −80°C for subsequent RNA extraction and lycopene measurements.

### Total RNA isolation, cDNA synthesis, DNA extraction, and PCR amplification

The 11 golden rules of qRT-PCR were observed in the experiments [24]. Total RNA was isolated from the frozen samples by following the TransZol (TransGen, China) protocol. The integrity of the extracted RNA was verified using 2% agarose gel electrophoresis. The concentration and purity of RNA were measured using NanoDrop 2000 spectrophotometer (Thermo Scientific, China). RNA samples with A_260_/A_280_ > 1.8 and A_260_/A_230_ > 2.0 were used for subsequent cDNA synthesis. Before cDNA synthesis, genomic DNA (gDNA) was eliminated using PrimeScript RT Reagent Kit with gDNA Eraser (Perfect Real Time) (TaKaRa, China) according to the manufacturer’s instructions. An equal amount of total RNA (1 μg) from each sample was used for each 20 μL reverse transcription reaction system. gDNA was extracted from the samples using Plant Genomic DNA Kit (Tiangen, China) and amplified using 2 × PCR Reagent (Tiangen, China). The amplified products were resolved by 2% agarose gel electrophoresis.

### Candidate reference gene selection and primer design

Reference genes that had been validated on other crops, particularly in fruits, were selected as candidates. The descriptions of these genes are provided in Table 1. For each candidate reference gene, BLASTN was conducted in the Cucurbit Genomics Database (http://www.icugi.org) against watermelon CDS (v1) by using *Arabidopsis* homolog as a query. The sequence of the best-matched gene with its structural information was downloaded. Primer3Plus (http://primer3plus.com/cgi-bin/dev/primer3plus.cgi) was employed for primer design. To control the potential gDNA contaminations in cDNA samples, one primer sequence was designed to cover an exon–exon junction. Then, the generated primer pairs were aligned against all watermelon CDS by Primer-BLAST (http://www.ncbi.nlm.nih.gov/tools/primer-blast/) to confirm its specificity on genome scale. PCR amplification specificity for each gene was further verified by 2% agarose gel electrophoresis, with gDNA and cDNA as templates. Finally, the watermelon species *Cl* was added as a prefix to the gene name to designate the watermelon orthologous gene. For more comparable results, the known primer sequences of *ClACT* and *18SrRNA*, which had been used as reference genes in watermelon, were used in this study [1, 17].

**Table 1:** Description of the candidate reference genes, primer sequences, and PCR amplification characteristics.

Gene Name	Gene description	Gene ID ^a^	Forward primer sequence (5’-3’)	Reverse primer sequence (5’-3’)	Product size (bp)	*E* (%)	*R* ^*2*^
*ClCAC*	Clathrin adaptor complex subunit	Cla016178	GAACTTGGCACCTGTCCTGT	GAACAGTGCAACAGCCTCAA	147	104.3	0.999
*ClPP2A*	Protein phosphatase 2A regulatory subunit A	Cla021905	GTGATTATGTGGACCGTGGA	TCCAGACATTTGCATTTCCA	180	95.9	1.000
*ClRAN*	A member of RAN GTPase gene family	Cla012277	TTGAAAAGAAATACGAACCAACC	ATCCCGTAAACCACCGAACT	128	103.0	0.999
*ClRPS15*	Cytosolic ribosomal protein S15	Cla021565	AAGCTGCGAAAAGCGAAAC	TAATGGCCAATCATCTCAGG	170	92.8	0.992
*ClSAND*	SAND family protein	Cla001870	TGCAAACATAAGGTTATCAGTCTTG	GCATACAAAAACGCCATAGGA	168	96.7	0.998
*ClTBP2*	TATA binding protein 2	Cla011119	CCAGAGTTATTCCCTGGATTG	CCTGGACATGCGCCTTAG	109	93.4	0.998
*ClTIP41*	TIP41-like family protein	Cla016074	CAAGCTCTCGCTGAAAAAGG	GAGACTCTGAGCTTTTGGGTTT	112	96.4	1.000
*ClTUA5*	Alpha tubulin 5	Cla003129	GATGGTATGATGCCCAGTGA	CCGGTAGGCTCCAGTTCTAA	156	100.2	1.000
*ClTUB*	β-tubulin	Cla022418	CAGCACTCCTAGCTTTGGTGA	CGGGGAAATGGGATTAGATT	136	96.9	0.999
*ClUPL7*	Ubiquitin-protein ligase 7	Cla017746	TGGCAAACGACATGTTATTGA	TTGAAAAAGGCAATATCTGTCG	128	105.7	1.000
*ClACT* ^b^	β-actin		CCATGTATGTTGCCATCCAG	GGATAGCATGGGGTAGAGCA		104.0	0.999
*Cl18SrRNA* ^b^	18SrRNA		AGCCTGAGAAACGGCTACCACATC	ACCAGACTCGAAGAGCCCGGTAT		94.8	0.998

^a^ Watermelon gene ID in the Cucurbit Genomics Database (http://www.icugi.org).

^b^ The primer pairs of *ClACT* previously published by Kong et al. [17] and *18SrRNA* previously published by Guo et al. [1] were used here.

### qRT-PCR analysis

Approximately 80 ng of cDNA was used as template for qRT-PCR analysis. cDNA samples were amplified by using 1 × Top Green qPCR SuperMix (TransGen, China) and 0.2 μM of each primer in a final volume of 10 μL. The reactions were performed on a LightCycler480 System (Roche, Switzerland) with two technical replicates for each of the three biological replicates described previously. The negative controls without templates were also included in each run. PCR cycling conditions consisted of an initial denaturation at 94°C for 30 s, followed by 40 cycles of denaturation at 95°C for 5 s, annealing at 55°C for 15 s, and extension at 72°C for 10 s. Melting cure analysis was performed at the end of amplification to verify its specificity. PCR amplification efficiency for each gene was determined by amplifying fivefold serial dilution of a mixture of all cDNA samples (from 800 ng μL^-1^ to 1.28 ng μL^-1^).

### Evaluation of expression stability

Expression levels depicted as crossing point (Cp) values were recorded for the tested reference genes after each qRT-PCR reaction. A boxplot was drawn using R package (http://www.r-project.org/) to show the expression variation of each gene. The amplification efficiency (E) was calculated using the following formula: E = (10^−1/slope^—1), where the slope is the standard curve slope generated by amplifying the fivefold serial dilution of pooled cDNA samples. NormFinder [16] and geNorm [15] were used to evaluate the gene expression stability. GeNorm calculated not only the gene expression stability value (M) for each gene, but also the pairwise variation values (V) to determine the minimum number of reference genes required for reliable normalization. No additional genes were required for normalization when the pairwise variation (*V*_*n/n+1*_) was below 0.15 [15]. Using a model-based approach, NormFinder considered the variations across groups and calculated the expression stability for each reference gene [16]. Lower stability values in both algorithms implied the higher expression stability of the genes. Given that the input data were supposed to be on a linear scale, the raw Cp values were corrected by PCR efficiency and converted into relative expression quantities *Q* by using the formula: *Q* = (1+ E)^(minCp-sampleCp)^ before data entry. To evaluate the effects of genotypes and fruit setting methods on the expression stability of the candidate reference genes, 48 diverse samples were further categorized into different subgroups according to the treatments during data analysis.

### Determination of *ClPSY1* expression and lycopene contents during fruit ripening

The samples derived from the pollinated fruit of 8424 at 10, 18, 23, 27, 30, and 35 DAA were used to analyze *ClPSY1* (Cla009122) expression patterns, which was associated with lycopene accumulation in watermelon fruit [5]. The primer sequences used for *ClPSY1* were F: 5’-CTAGCAGATGGCCGGTGT-3’ and R: 5’-GCCCTCTTTGTGAAGTTGTTG-3’, which were designed and validated according to the methods mentioned above. The top-ranked reference genes identified in this study, as well as *18SrRNA*, were used for the normalization. The relative expression levels of *ClPSY1* were evaluated using 2^−△△Cp^ method. Normalization factor was calculated as the geometric mean of two or multiple reference genes and used for normalization. The fruit flesh was still white in color at 10 DAA but turned to pink at 18 DAA. Accordingly, the lycopene contents were measured at 18, 23, 27, 30, and 35 DAA. Extraction of carotenoids and measurement of lycopene contents were conducted as described by Liu et al. [25]. Waters 1525 reversed phase HPLC equipped with 2996 photodiode array detector, 717 plus autosampler, Empower Chromatography Manager software (Waters, USA), and YMC C30 carotenoid column (150 mm × 4.6 mm i.d., 3 µm, YMC, USA) was used for lycopene detection and quantification. HPLC-grade lycopene standard was obtained from Sigma-Aldrich (Shanghai, China). Three biological and two technical replicates were adopted in qRT-PCR analysis and lycopene content measurement for each sample.

## Results

### Amplification specificity and efficiency of candidate reference genes

**Fig 1:**
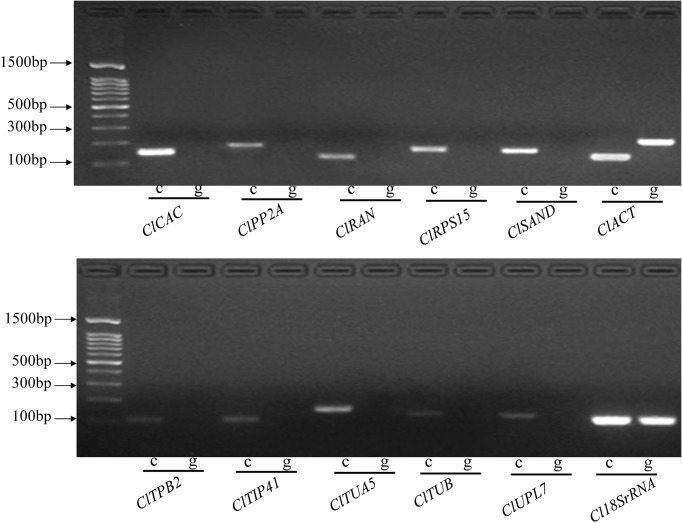
PCR amplification products of the candidate reference genes on cDNA and gDNA templates. “c” represents the cDNA template. “g” represents the gDNA template.

The candidate reference genes included 10 reference genes that were previously validated in fruits of other crops, *Cl18SrRNA*, and *ClACT*. To prevent the interferences of potential gDNA contaminations in cDNA samples on the expression analysis, one primer was designed to cover an exon–exon junction for each gene. For more comparable results, primer pairs of *Cl18SrRNA* and *ClACT* published previously were also used in this study. PCR-amplification specificity of each primer pair was verified by agarose gel electrophoresis using cDNA and gDNA templates. Each reference gene amplified a specific product on cDNA templates, but no products were detected on gDNA templates except for *ClACT* and *Cl18SrRNA*, demonstrating the success of primer design (Fig 1). Moreover, *ClACT* amplified a larger band containing an intron on gDNA, but not on cDNA, indicating the absence of gDNA contamination in cDNA samples. *Cl18SrRNA* amplified the same bands on both gDNA and cDNA templates. A single peak with no visible primer–dimer formation was observed in melting-curve analysis, further confirming the specific amplification of each reference gene (S1 Fig). No signals were detected in the controls without templates in qRT-PCR reactions. PCR-amplification efficiencies of the candidate genes ranged from 92.8% (*ClRPS15*) to 105.7% (*ClUPL7*). The determination coefficients (*R* ^*2*^) of the standard curve regression equation varied from 0.992 (*ClRPS15*) to 1.000 (*ClPP2A*). These parameters indicated that qRT-PCR systems established for the candidate reference genes were highly specific and efficient. The primer sequences and amplification characteristics of the candidate reference genes are presented in Table 1.

### Expression patterns of candidate reference genes

**Fig 2:**
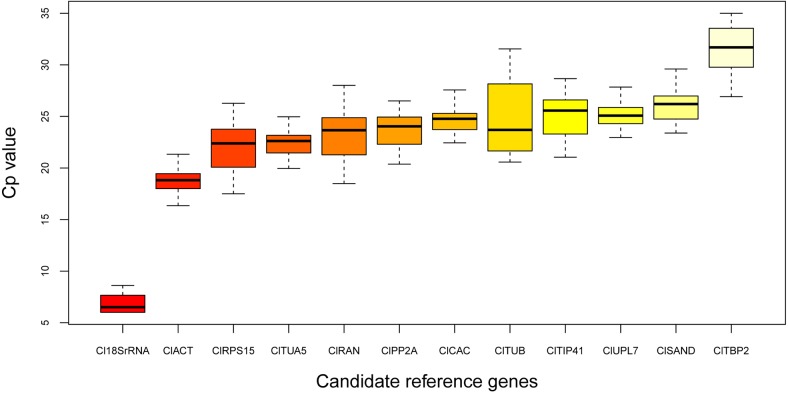
Boxplot analysis of expression variations of the tested reference genes in all 48 samples. The line across the box is the median. The boxes are 25/75 percentiles. Whisker caps are the minimum and maximum values.

Similar to artificial pollination, 100% fruit set was obtained with CPPU treatment. Pollinated fruits had normal seeds, whereas CPPU treatments resulted in the development of seedless fruits. Expression profiles of the 12 candidate reference genes in watermelon fruits were obtained by qRT-PCR analysis. Expression variations among the two genotypes and two fruit setting methods in 12 successive development stages were found (Fig 2). The mean Cp values for the tested genes varied from 6.8 (*Cl18SrRNA*) to 31.9 (*ClTBP2*) and were mainly between 21.0 and 26.0. *Cl18SrRNA*, *ClUPL7*, and *ClTUA5* exhibited smaller expression variations among the selected reference genes (< 5 cycles), whereas *ClTBP2*, *ClRPS15*, *ClRAN*, and *ClTUB* demonstrated relatively higher expression variations (> 8 cycles). Direct comparison of raw Cp values could not accurately estimate the expression stability of each reference gene because of the existence of non-biological variations in experimental processes. Consequently, statistical approaches were required to evaluate the expression stability of the candidate reference genes.

### Expression stability of candidate reference genes

The widely used tools of geNorm and NormFinder were used to assess the expression stability of the tested genes. Considering the effects of genotypes, fruit setting methods and developmental phases on the 48 samples, geNorm determined that *ClCAC* and *ClACT* was the best pair of reference genes (Table 2). Pairwise variation analysis showed that at least three reference genes, including *ClCAC*, *ClACT*, and *ClTUA5*, were required for more reliable normalization when the proposed cut-off value of 0.15 was adopted (S2 Fig). To study the effects of genotype and fruit setting method on expression stability of the reference genes and to identify more stable reference genes under specific fruit development conditions, 48 samples were further divided into different subgroups according to the treatments listed in Table 2. In the inbred line 97103, the best pairs of reference genes for pollination and CPPU treatments were *ClSAND* and *ClACT*; and *ClCAC* and *ClACT*. In F_1_ hybrid 8424, *ClCAC* and *ClACT* were ranked as the best pair of genes regardless of fruit setting methods; they were also the best pair of genes for different fruit setting treatments regardless of genotypes. However, pairwise variations showed that different minimum numbers of reference genes (from 2 to 4) were required for more accurate normalization in the different subgroups (Table 2, S2 Fig). *ClTUB* was identified as the least stable gene under all conditions.

**Table 2:** Expression stability of the candidate reference genes determined by geNorm.

Ranking			97103				8424				97103 and 8424			
	All samples	M *n = 48* ^*a*^	Pollination	M *n = 12*	CPPU treatment	M *n = 12*	Pollination	M *n = 12*	CPPU treatment	M *n = 12*	Pollination	M *n = 24*	CPPU treatment	M *n = 24*
1	*ClCAC* ^*b*^	0.41	*ClSAND* ^*b*^	0.21	*ClCAC* ^*b*^	0.26	*ClCAC* ^*b*^	0.26	*ClCAC* ^*b*^	0.21	*ClCAC* ^*b*^	0.46	*ClCAC* ^*b*^	0.35
1	*ClACT* ^*b*^	0.41	*ClACT* ^*b*^	0.21	*ClACT* ^*b*^	0.26	*ClACT* ^*b*^	0.26	*ClACT* ^*b*^	0.21	*ClACT* ^*b*^	0.46	*ClACT* ^*b*^	0.35
2	*ClTUA5* ^*b*^	0.53	*ClCAC*	0.31	*ClTUA5* ^*b*^	0.41	*ClTUA5* ^*b*^	0.4	*ClPP2A*	0.37	*ClUPL7* ^*b*^	0.56	*ClTUA5* ^*b*^	0.45
3	*ClUPL7*	0.58	*ClPP2A*	0.34	*ClPP2A*	0.48	*ClUPL7*	0.46	*ClSAND*	0.41	*ClTUA5* ^*b*^	0.62	*ClPP2A*	0.51
4	*ClSAND*	0.63	*ClTIP41*	0.41	*ClUPL7*	0.56	*ClSAND*	0.6	*ClTUA5*	0.42	*ClSAND*	0.66	*ClSAND*	0.56
5	*ClPP2A*	0.67	*ClRPS15*	0.45	*ClSAND*	0.62	*ClPP2A*	0.68	*ClUPL7*	0.48	*ClPP2A*	0.74	*ClUPL7*	0.6
6	*ClTIP41*	0.78	*ClUPL7*	0.48	*ClTIP41*	0.7	*ClTBP2*	0.76	*ClTIP41*	0.63	*ClTIP41*	0.85	*ClTIP41*	0.7
7	*ClRPS15*	0.86	*ClTUA5*	0.56	*ClRPS15*	0.78	*ClTIP41*	0.91	*ClTBP2*	0.75	*ClRPS15*	0.92	*ClRPS15*	0.8
8	*ClRAN*	0.97	*ClRAN*	0.62	*ClRAN*	0.85	*ClRPS15*	1.03	*ClRPS15*	0.85	*ClRAN*	1.01	*ClRAN*	0.92
9	*Cl18SrRNA*	1.08	*Cl18SrRNA*	0.69	*Cl18SrRNA*	0.95	*ClRAN*	1.12	*ClRAN*	0.94	*Cl18SrRNA*	1.12	*Cl18SrRNA*	1.04
10	*ClTBP2*	1.21	*ClTBP2*	1	*ClTBP2*	1.1	*Cl18SrRNA*	1.26	*Cl18SrRNA*	1.08	*ClTBP2*	1.27	*ClTBP2*	1.15
11	*ClTUB*	1.41	*ClTUB*	1.22	*ClTUB*	1.37	*ClTUB*	1.44	*ClTUB*	1.3	*ClTUB*	1.45	*ClTUB*	1.38

^a^ The sample size.

^b^ The multiple reference genes determined by pairwise variation analysis when the cut-off value 0.15 was adopted (S2 Fig).

NormFinder is more powerful in detecting inter- and intra-group variations. Therefore, the samples were divided into different subgroups based on genotypes and fruit setting methods in calculations. The results are shown in Table 3. Considering the four subgroups of two genotypes and their respective fruit setting methods, NormFinder ranked *ClCAC* as the single best reference gene and *ClCAC* and *ClSAND* as the combination of two best genes. The effects of genotype and fruit setting method on the expression stability of the candidate reference genes were also evaluated. *ClCAC* alone or together with *ClRAN* was ranked as the single best reference gene or the combination of two best genes during fruit development of 97103. Similarly, *ClSAND* alone or together with *ClCAC* was ranked as the single or two best reference genes during fruit development of 8424 regardless of fruit setting methods. During the development of pollinated fruits, which included genotypes 97103 and 8424, *ClCAC* was the single best reference gene, whereas *ClPP2A* and *ClACT* were the two best genes. By contrast, *ClSAND* alone or together *ClCAC* were ranked as the single or the two best reference genes in the parthenocarpic fruit induced by CPPU treatment. *ClTBP2* and *ClTUB* were ranked as the least stable genes under different conditions.

**Table 3:** Expression stability of the candidate reference genes determined by NormFinder.

Ranking	All samples (*n = 48* ^*a*^)		97103 (*n = 24*)		8424 (*n = 24*)		Pollination (*n = 24*)		CPPU treatment (*n = 24*)	
	Gene name	M	Gene name	M	Gene name	M	Gene name	M	Gene name	M
1	*ClCAC* ^b^	0.16	*ClCAC* ^b^	0.11	*ClSAND* ^b^	0.09	*ClCAC*	0.14	*ClSAND* ^b^	0.11
2	*ClSAND* ^b^	0.18	*ClACT*	0.17	*ClCAC* ^b^	0.16	*ClPP2A* ^b^	0.15	*ClCAC* ^b^	0.16
3	*ClACT*	0.22	*ClRAN* ^b^	0.17	*ClTUA5*	0.17	*ClSAND*	0.2	*ClACT*	0.19
4	*ClPP2A*	0.3	*ClTIP41*	0.18	*ClACT*	0.2	*ClACT* ^b^	0.27	*ClTIP41*	0.21
5	*ClTIP41*	0.32	*ClRPS15*	0.2	*ClPP2A*	0.22	*ClTUA5*	0.34	*ClUPL7*	0.27
6	*ClTUA5*	0.34	*ClSAND*	0.22	*ClTIP41*	0.26	*ClUPL7*	0.36	*ClPP2A*	0.3
7	*ClRPS15*	0.41	*ClTUA5*	0.24	*ClRAN*	0.28	*ClTIP41*	0.39	*ClRPS15*	0.32
8	*ClUPL7*	0.42	*ClUPL7*	0.29	*ClUPL7*	0.29	*Cl18SrRNA*	0.41	*ClTUA5*	0.33
9	*Cl18SrRNA*	0.46	*ClPP2A*	0.32	*ClRPS15*	0.29	*ClRPS15*	0.53	*ClRAN*	0.42
10	*ClRAN*	0.49	*Cl18SrRNA*	0.32	*ClTBP2*	0.3	*ClRAN*	0.57	*ClTBP2*	0.46
11	*ClTUB*	0.62	*ClTBP2*	0.45	*Cl18SrRNA*	0.42	*ClTUB*	0.6	*Cl18SrRNA*	0.46
12	*ClTBP2*	0.73	*ClTUB*	0.55	*ClTUB*	0.49	*ClTBP2*	1.08	*ClTUB*	0.61

^a^ The sample size.

^b^ The combination of two best genes.

*Cl18SrRNA*, frequently used as reference gene in watermelon fruit, was ranked both by geNorm and NormFinder as second to fifth from the bottom in all samples and different subsets, indicating that this gene was unsuitable for normalization in qRT-PCR analysis in watermelon fruit.

### Validation of top-ranked reference genes

**Fig 3:**
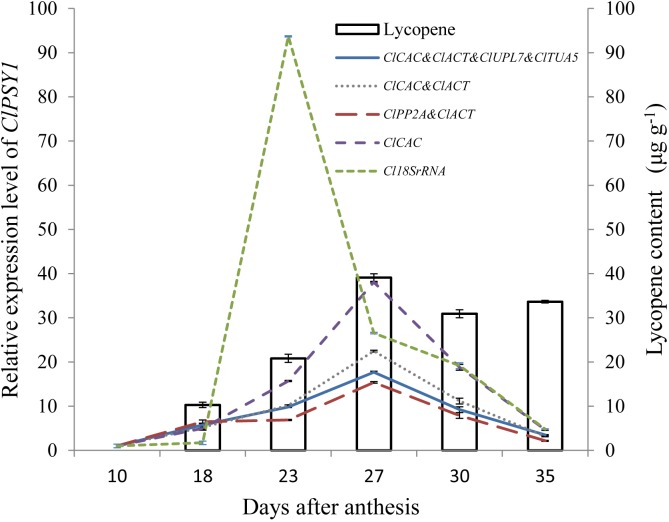
Lycopene accumulation and expression profiles of *ClPSY1* during fruit ripening. Geometric mean was calculated for the two or multiple reference genes, and used for normalization. *Cl18SrRNA* was used as control. The results are depicted as mean ± SE (*n* = 6).

To validate the reliability of the top-ranked reference genes, expression pattern of *ClPSY1* and lycopene accumulation were analyzed in the pollinated fruits of 8424. The single (*ClCAC* as determined by NormFinder), pair (*ClCAC* and *ClACT* as determined by geNorm, and *ClPP2A* and *ClACT* as determined by NormFinder), and multiple best reference genes (*ClCAC*, *ClACT*, *ClUPL7*, and *ClTUA5* as determined by geNorm) identified in the pollination subset regardless of genotypes were used to normalize the expressions of *ClPSY1*. *Cl18SrRNA*, the widely used reference gene in watermelon fruit, was used as the control. The results are presented in Fig 3.

*ClPSY1* was expressed at 10 DAA with the raw Cp value of 24.4. Thus, the transcript abundance of *ClPSY1* at 10 DAA was set as the control. By using the top-ranked reference genes for normalization, the same *ClPSY1* expression trends were observed, gradually increasing after 10 DAA, peaking at 27 DAA, and then decreasing (Fig 3). By contrast, different *ClPSY1* expression levels were observed using different top-ranked reference genes. The highest expression level was observed using the best single reference gene *ClCAC*, whereas the lowest expression level was obtained using the combination of *ClPP2A* and *ClACT* as determined by NormFinder. Moderate expression levels were found using the pair and multiple reference genes as determined by geNorm, with only slight difference on expression levels noted between the two observations. However, using *Cl18SrRNA* for normalization, distinct *ClPSY1* expression pattern was observed, which remained stable from 10 DAA to 18 DAA, sharply increasing to the highest level at 23 DAA and then decreased as the fruit matured.

Lycopene accumulation was measured from 18 DAA, whereas the fruit flesh was white at 10 DAA. The results showed that lycopene content gradually increased as the fruit ripened and reached the highest level of 39.08 μg·g^−1^ fresh weight at 27 DAA and then decreased and remained stable from 30 DAA to 35 DAA. Good correlations were found between lycopene biosynthesis until 27 DAA and *ClPSY1* expression by using the top-ranked reference genes for normalization. However, when normalized by *18SrRNA*, no correlation was observed between *ClPSY1* expression pattern and lycopene accumulation in ripening fruit.

## Discussion

Accurate interpretation of qRT-PCR results mainly depends on the use of stable reference genes for data normalization to minimize non-biological variations between samples. Consequently, systematic validation of reference genes is essential to produce reliable data in qRT-PCR analysis [13, 19, 24]. At present, systematic validation of reference genes is always conducted across a wide range of organ samples in different experimental conditions [17, 26]. However, increasing evidence shows that the best reference genes varied significantly depending on experimental conditions and organs assayed; compiling a list of suitable genes that could be used as references across a wide range of experimental conditions was impossible [19, 27]. Therefore, validating references under specific experimental condition is essential. To date, expression studies performed on watermelon fruits have used only *18SrRNA* for normalization [1, 2, 22]. No systematic study has been carried out to verify the expression stability of the reference gene used in watermelon fruit or to identify more reference genes. Thus, screening and validation of appropriate reference genes specifically for watermelon fruit are urgently needed to facilitate the accurate expression measurement of target genes.

In this study, systematic validation of optimal reference genes was conducted for watermelon fruit considering the effects of fruit setting methods, genotypes, and fruit developmental stages. Although many algorithms have been developed to aid in the selection of appropriate reference genes, geNorm and NormFinder proved to be sufficient for validation of reference genes [20]. Considering all 48 samples, geNorm determined *ClCAC* and *ClACT* as the best pair of reference genes. *ClCAC*, *ClACT*, and *ClTUA5* were also required to calculate a reliable normalization factor. NormFinder ranked *ClCAC* alone or together with *ClSAND* as the single or two best genes. Different results were expected because of the different algorithms adopted in the two methods. GeNorm is based on the principle that the expression ratio between two ideal internal control genes is identical in all samples regardless of the experimental condition [15]. By contrast, NormFinder is a model-based approach that estimates expression variations and ranks the candidate reference genes with minimal estimated intra- and inter-group variations [16]. In this study, all 48 samples were divided into different subgroups according to genotypes and fruit setting methods to comply with the model-based algorithm of NormFinder. Consequently, NormFinder potentially provided a more robust measurement of gene expression stability [16]. Meanwhile, *CAC* and *SAND* were also recommended in tomato fruit [28]. Suitable reference genes were also systemically validated in fruits in other studies. However, different results were obtained in different species. For example, *CAC* and *UBQ2* were the most stable genes in banana pulp [29]. In papaya fruit, *EIF*, *TBP1*, and *TBP2* exhibited good performance [20]. *ACT* was recommended in lychee fruit [30]. These results demonstrate that screening for appropriate reference genes is a prerequisite before qRT-PCR analysis. However, the reference genes identified in other studies were good candidates, and verifying the stability of these candidates was an efficient approach to find the most suitable references [31, 32].

After dividing the samples into different groups according to the treatments and calculating the expression stability of the candidate genes, differences on the rankings of the candidate reference genes were observed between different genotypes and fruit setting methods. In genotype 97103, geNorm ranked *ClSAND* and *ClACT* as well as *ClCAC* and *ClACT* as the best pairs of reference genes for the pollination and CPPU treatments. Although, the best pair of reference genes was *ClCAC* and *ClACT* for both pollination and CPPU treatment in 8424, the minimum number of reference genes to calculate the reliable normalization factor was 4 and 3, respectively. When the data from the two genotypes were integrated, the minimum number of reference genes required to calculate the normalization factor was different (Table 2). Based on the results determined by NormFinder, the differences on the single best gene or combination of two best genes were more pronounced between watermelon genotypes or fruit setting methods (Table 3). Thus, both the genotypes and fruit setting methods affected the rankings of the candidate references. The suitable reference genes were also found to be genotype dependent in several crops, such as mustard [12], grapevine [33], strawberry [34], and sweet potato [35]. These findings partially explained why different best reference genes were obtained in different experiments on the same crop. As a result, appropriate reference genes should be validated or selected under specific experimental conditions. However, the validation of reference genes is very tedious and impossible to conduct before each experiment, particularly when the number of target genes to be normalized is limited. Thus, specialized studies on reference gene validation are necessary to test the suitability of candidate reference genes as many as possible. To obtain representative and widely applicable results, the experiments should be designed to include the affecting factors as many as possible [34]. In this study, aside from the fruit developmental stages, the effects of genotypes and fruit setting methods were also considered and evaluated, which nearly included all the potential factors that affect watermelon fruit development under normal growth condition, to provide a comprehensive evaluation of the expression stability of the selected reference genes.

Transcriptional regulation has shown to be a major mechanism that controls the biosynthesis of specific carotenoids [5]. *ClPSY1* is one of the key genes that control lycopene biosynthesis in watermelon fruits [1, 5]. Thus, the correlation of *ClPSY1* expression pattern and lycopene biosynthesis during the ripening of watermelon fruit can offer a reliable measurement to test the reliability of top-ranked reference genes, and good correlations were found between lycopene accumulation and *ClPSY1* expression by using the top-ranked reference genes for normalization. However, when normalized by *Cl18SrRNA*, no correlation was observed between *ClPSY1* expression pattern and lycopene biosynthesis in fruit ripening. These results demonstrate that the top-ranked reference genes in this study are suitable for normalization during watermelon fruit development.

Nevertheless, different expression levels of *ClPSY1* were observed using different top-ranked reference genes. The highest expression level was observed by using *ClCAC*, whereas the lowest expression level was obtained by using *ClPP2A* and *ClACT* as determined by NormFinder. Moderate expression levels were found using the pair and multiple reference genes as determined by geNorm. Similar results were also observed in previous studies [17, 36]. Considering that the variation in the average of multiple genes was smaller than the variation in individual genes, the normalization factor calculation based on the geometric mean of multiple reference genes provided more accurate and reliable normalization of expression data [15, 16]. Accordingly, the multiple reference genes identified in this study were recommended to replace the single and non-validated *18SrRNA* for more reliable normalization results in watermelon fruit.

Considering the significant effect of reference genes on the normalization results, primer specificities of the candidate reference genes have been stressed in many studies [16, 24]. Reference genes with primers spanning the exon–exon junction were suggested to control the potential gDNA contamination [37]. Meanwhile, primer pairs flanking an intron were also designed for candidate reference genes [16, 17, 38]. In this study, the primers spanning the exon–exon junction were developed for 10 selected candidate reference genes, along with *ClACT* with amplicon spanning an intron developed in our previous study [17]. In practice, *ClACT* can be used first to test the existence of gDNA contamination in cDNA samples. In the absence of gDNA contaminations, both the multiple reference genes of *ClCAC*, *ClACT*, and *ClTUA5* and the best combination of *ClCAC* and *ClSAND* can be used for normalization. However, in the presence of gDNA contaminations, the best combination of *ClCAC* and *ClSAND* is preferred for normalization if the primers of the target genes also spanned the exon–exon junction. Thus, these reference genes provided a powerful strategy for normalization in qRT-PCR analysis for watermelon fruits.

## Conclusions

In this study, the major factors including genotypes, fruit setting methods, and fruit developmental stages that potentially affect the reference gene expression stability in watermelon fruits have been considered to develop reliable and widely applicable results. The normalization factor calculated from the geometric means of *ClCAC*, *ClACT*, and *ClTUA5* as determined by geNorm or *ClCAC* and *ClSAND* as identified by NormFinder can be used to normalize the target genes in watermelon fruit to improve the accuracy and reliability of gene expression studies. Moreover, this study provides a list of potential reference genes with specific primers spanning exon–exon junction that can be easily tested in other reference gene studies for watermelon.

## Supporting Information

S1 Fig Amplification specificity of the primer pairs verified by melting curve analysis. (PDF)

S2 Fig Pairwise variation analyses of the candidate reference genes by geNorm. (PDF)
