# Supplementary material for: Evaluation of Appropriate Reference Genes for Gene Expression Normalization during Watermelon Fruit Development
Source: PLoS One. 2015 Jun 25;10(6):e0130865. doi: 10.1371/journal.pone.0130865 (PMC4481515; doi:10.1371/journal.pone.0130865)
Supplement: S1 Fig — (PDF) [file pone.0130865.s001.pdf]

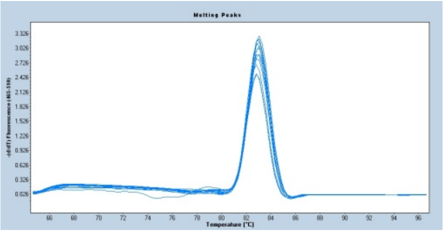

ClACT

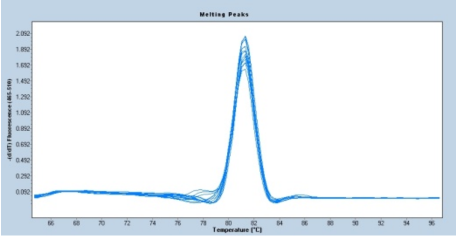

ClCAC

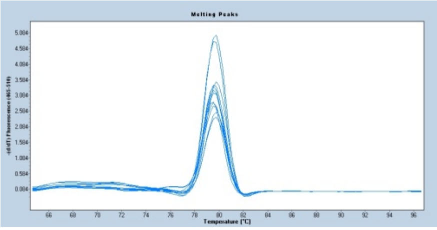

ClPP2A

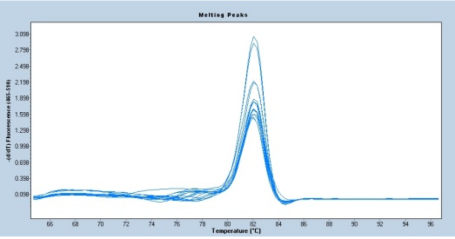

ClRAN

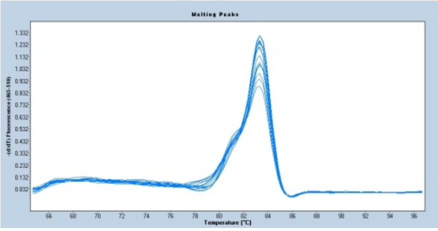

ClRPS15

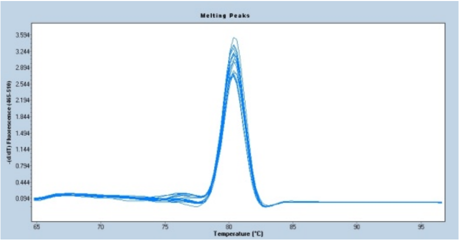

ClSAND

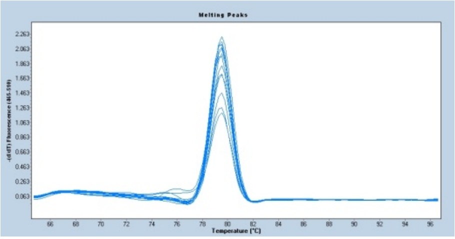

ClTBP2

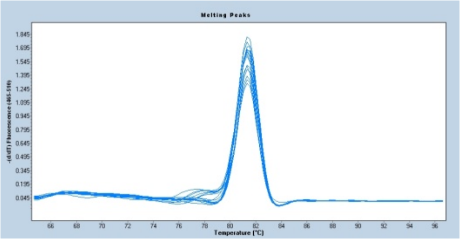

ClTIP41

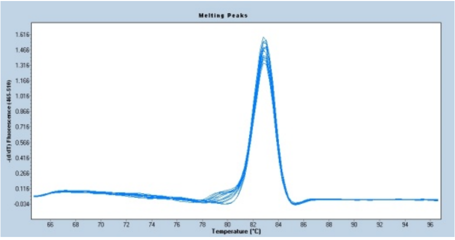

ClTUA5

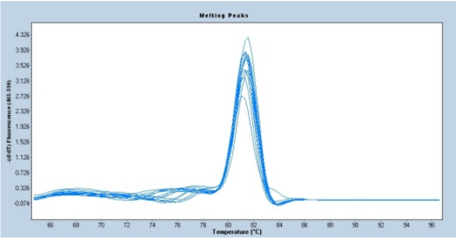

ClTUB

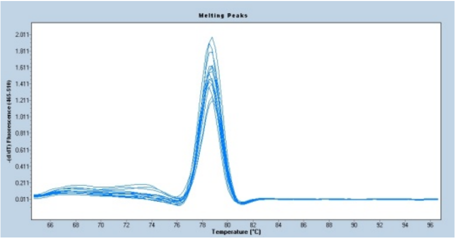

ClUPL7

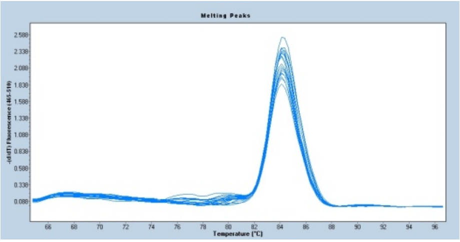

Cl18SrRNA

S1 Fig. Amplification specificity of the primer pairs verified by melting curve analysis
