# Supplementary material for: Evaluation of Appropriate Reference Genes for Gene Expression Normalization during Watermelon Fruit Development
Source: PLoS One. 2015 Jun 25;10(6):e0130865. doi: 10.1371/journal.pone.0130865 (PMC4481515; doi:10.1371/journal.pone.0130865)
Supplement: S2 Fig — (PDF) [file pone.0130865.s002.pdf]

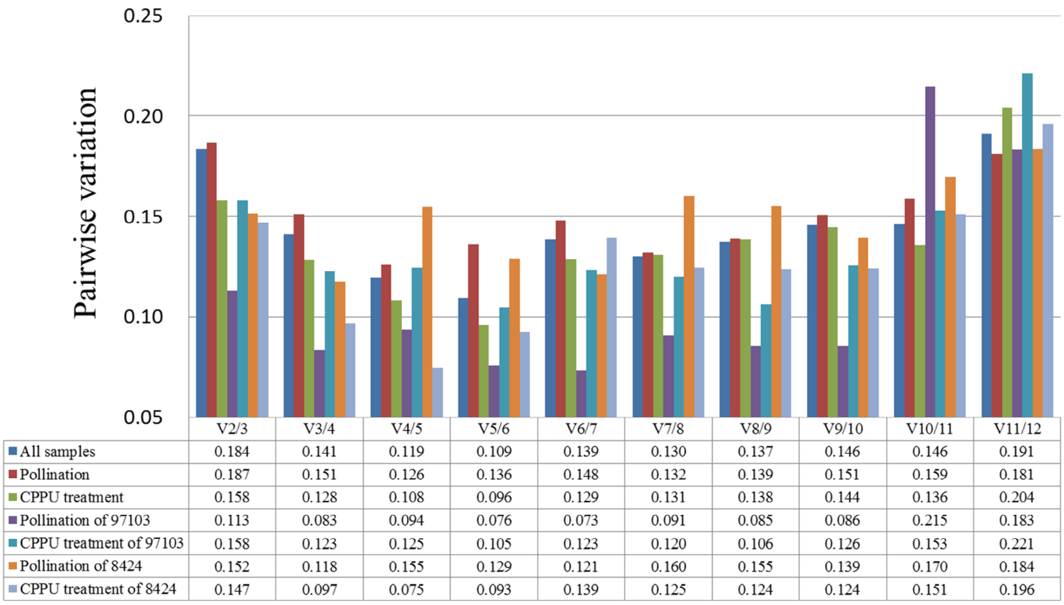

	V2/3	V3/4	V4/5	V5/6	V6/7	V7/8	V8/9	V9/10	V10/11	V11/12
All samples	0.184	0.141	0.119	0.109	0.139	0.130	0.137	0.146	0.146	0.191
Pollination	0.187	0.151	0.126	0.136	0.148	0.132	0.139	0.151	0.159	0.181
CPPU treatment	0.158	0.128	0.108	0.096	0.129	0.131	0.138	0.144	0.136	0.204
Pollination of 97103	0.113	0.083	0.094	0.076	0.073	0.091	0.085	0.086	0.215	0.183
CPPU treatment of 97103	0.158	0.123	0.125	0.105	0.123	0.120	0.106	0.126	0.153	0.221
Pollination of 8424	0.152	0.118	0.155	0.129	0.121	0.160	0.155	0.139	0.170	0.184
CPPU treatment of 8424	0.147	0.097	0.075	0.093	0.139	0.125	0.124	0.124	0.151	0.196

S2 Fig. Pairwise variation analyses of the candidate reference genes by geNorm. Pairwise variation was calculated by geNorm to determine the minimum number of reference genes required for accurate normalization in different sample sets. The Pollination subgroup includes the samples from the pollinated fruits of 97103 and 8424. Similarly, the CPPU treatment subgroup includes the samples from the CPPU treated fruits of 97103 and 8424. The grouping method is the same as in Table 2.
